# Electrophysiological properties of myocytes isolated from the mouse atrioventricular node: L-type *I*_C__a_, *I*_K__r_, *I*_f_, and Na-Ca exchange

**DOI:** 10.14814/phy2.12633

**Published:** 2015-11-25

**Authors:** Stéphanie C Choisy, Hongwei Cheng, Clive H Orchard, Andrew F James, Jules C Hancox

**Affiliations:** School of Physiology and Pharmacology and Cardiovascular Research Laboratories, Biomedical Sciences Building, University of BristolBristol, United Kingdom

**Keywords:** atrioventricular node, AV node, AVN, calcium-current, hyperpolarization-activated current, *I*_C__a,L_, *I*_f_, *I*_K__r_, *I*_NCX_, pacemaking, rapid delayed rectifier, ryanodine

## Abstract

The atrioventricular node (AVN) is a key component of the cardiac pacemaker-conduction system. This study investigated the electrophysiology of cells isolated from the AVN region of adult mouse hearts, and compared murine ionic current magnitude with that of cells from the more extensively studied rabbit AVN. Whole-cell patch-clamp recordings of ionic currents, and perforated-patch recordings of action potentials (APs), were made at 35–37°C. Hyperpolarizing voltage commands from −40 mV elicited a Ba^2+^-sensitive inward rectifier current that was small at diastolic potentials. Some cells (Type 1; 33.4 ± 2.2 pF; *n* = 19) lacked the pacemaker current, *I*_f_, whilst others (Type 2; 34.2 ± 1.5 pF; *n* = 21) exhibited a clear *I*_f_, which was larger than in rabbit AVN cells. On depolarization from −40 mV L-type Ca^2+^ current, *I*_C__a,L_, was elicited with a half maximal activation voltage (*V*_0.5_) of −7.6 ± 1.2 mV (*n* = 24). *I*_C__a,L_ density was smaller than in rabbit AVN cells. Rapid delayed rectifier (*I*_K__r_) tail currents sensitive to E-4031 (5 μmol/L) were observed on repolarization to −40 mV, with an activation *V*_0.5_ of −10.7 ± 4.7 mV (*n* = 8). The *I*_K__r_ magnitude was similar in mouse and rabbit AVN. Under Na-Ca exchange selective conditions, mouse AVN cells exhibited 5 mmol/L Ni-sensitive exchange current that was inwardly directed negative to the holding potential (−40 mV). Spontaneous APs (5.2 ± 0.5 sec^−1^; *n* = 6) exhibited an upstroke velocity of 37.7 ± 16.2 V/s and ceased following inhibition of sarcoplasmic reticulum Ca^2+^ release by 1 μmol/L ryanodine, implicating intracellular Ca^2+^ cycling in murine AVN cell electrogenesis.

## Introduction

The atrioventricular node (AVN) is an important component of the conduction system in mammalian hearts, with slow AVN conduction playing a key role in the normal sequence of atrial-ventricular excitation and contraction (Tawara 1906; Childers 1977; Meijler and Janse 1988). The AVN is normally the only route for excitation to pass from atria to ventricles and the slow conduction properties of the AVN can protect the ventricles from high atrial rates sustained during supraventricular tachycardias (SVTs) (Childers 1977; Selzer 1982; Meijler and Janse 1988). Under some conditions, however, the AVN can become part of the conduction circuit sustaining SVTs (Childers 1977; Meijler and Janse 1988; Nikolski et al. 2003). The AVN can also act as an accessory pacemaker of the ventricles in the event of failure of the primary pacemaker, the sinoatrial node (SAN) (Childers 1977; Meijler and Janse 1988). The site of origin of AVN rhythm has been proposed to involve the inferior (posterior) nodal extension of the AVN that has morphological and molecular properties similar to the compact node (Watanabe and Watanabe 1994; Dobrzynski et al. 2003; Li et al. 2008; Inada et al. 2009).

As for other areas of the heart, information on the electrophysiological properties of the AVN has come from both intact tissue preparations (e.g. (Meijler and Janse 1988; Billette 1987; Billette and Metayer 1989; Sun et* *al. 1995; Efimov et* *al. 2004)) and isolated AVN cell preparations. The majority of the available data on ionic currents underlying AVN activity comes from rabbit AVN cell and tissue preparations, both because the rabbit heart is sufficiently large to enable the AVN region to be excised reliably in order to isolate single cells and because a number of important intact AVN preparation electrophysiology studies have employed this species (reviewed in [Meijler and Janse 1988]). Initial studies identifying ionic currents important for AVN cell activity were performed on small tissue or “rounded” rabbit single AVN cell preparations (i.e., cell preparations that retained electrical activity, but lost their normal morphology on exposure to physiological bathing solutions) (Kokubun et* *al. 1980, 1982; Noma et* *al. 1980, 1984; Kurachi et* *al. 1981; Taniguchi et* *al. 1981; Nakayama et* *al. 1984; Nakayama and Irisawa 1985). Subsequent studies from the early 1990s onwards employed methods for isolating rabbit AVN cells that retained both normal cellular electrophysiology and morphology (e.g. [Hancox et* *al. 1993; Hancox and Levi 1994a,b; Martynyuk et* *al. 1995, 1996; Munk et* *al. 1996; Guo and Noma 1997; Workman et* *al. 1999; Mitcheson and Hancox 1999; Convery and Hancox 2000; Ren et* *al. 2006; Ridley et* *al. 2008; Cheng et* *al. 2009]). In 2009, the first biophysically detailed model of the AVN was published, based on experimental data from the rabbit AVN (Inada et* *al. 2009). In order to identify and distinguish general and species-specific properties, however, it is desirable to have AVN cellular electrophysiology data from additional model species. One study of rat AVN cells (Yuill et* *al. 2010) has shown properties broadly similar to those of rabbit AVN cells. Another study of adult guinea-pig AVN cells (Yuill and Hancox 2002) showed properties similar to those in rabbit, but also some differences: notably the presence of two components of delayed rectifier K^+^ current (*I*_Kr_ and *I*_Ks_) compared with only *I*_Kr_ in rabbit (Habuchi et* *al. 1995; Howarth et* *al., 1996), and the presence of a Ba^2+^-sensitive inwardly rectifying current at voltages negative to the diastolic potential range.

Due to its genetic tractability, relatively short breeding and life cycle, and low cost, the mouse offers a potentially valuable model for the study of the influence of gene expression and development on AVN electrophysiology. In recent years, some studies have utilized cells from the mouse AVN (e.g. [Zhang et* *al. 2008, 2011; Marger et* *al. 2011a,b]), demonstrating the feasibility of isolating and working with AVN myocytes from mouse hearts. Marger and colleagues have provided the most comprehensive information currently available on overall ionic current properties of mouse AVN cells, including data on calcium and potassium currents and on the hyperpolarization-activated current, *I*_f_ (Marger et* *al. 2011b). In the present study, we have sought to confirm and extend aspects of information available on murine AVN cellular electrophysiology. Specifically, in order to facilitate comparison with the data on the more extensively studied rabbit AVN, we used protocols previously used in the study of the rabbit AVN (Nakayama et* *al. 1984; Hancox et* *al. 1993; Hancox and Levi 1994b; Convery and Hancox 2000; Cheng et* *al. 2009; Choisy et* *al. 2012) to characterize selected currents from mouse AVN cells. Specifically, the use of similar holding (−40 mV) and step potentials (to a range of voltages in 10 mV increments) in prior rabbit AVN cell studies enabled a focus: (1) on currents including the hyperpolarization-activated pacemaker current *I*_f_, present over a range of voltages encompassing and extending beyond the diastolic potential range; (2) on *I*_Ca,L_, which in rabbit AVN cells is important for the rising phase of AVN APs (Nakayama et* *al. 1984; Hancox and Levi 1994a; Cheng et* *al. 2009; Choisy et* *al. 2012); (3) on rapid delayed rectifier current, *I*_Kr_, which is important both for AVN AP repolarization and pacemaker depolarization (Shibasaki 1987; Howarth et* *al., 1996; Mitcheson and Hancox 1999; Sato et* *al. 2000). Additionally, given accumulating evidence for a role of intracellular Ca^2+^ cycling in influencing AVN pacemaking (Hancox et* *al. 1994; Nikmaram et* *al. 2008; Ridley et* *al. 2008; Cheng et* *al. 2011, 2012), we measured for the first time Na-Ca exchange current *I*_NCX_ from mouse AVN cells and investigated the effect on spontaneous action potentials of sarcoplasmic reticulum inhibition with ryanodine.

## Materials and Methods

### Rabbit AVN cell isolation

Our long-established method for isolating rabbit AVN cells has been described previously (Hancox et* *al. 1993; Hancox and Levi 1994b; Cheng et* *al. 2009). Briefly, adult male New Zealand White rabbits (2–4 kg) were killed in accordance with UK Home Office regulations; the heart was then excised rapidly and perfused retrogradely at 37°C sequentially with calcium-containing, calcium-free and enzyme-containing (collagenase + protease) solutions (for further details of solutions see [Hancox et* *al. 1993; Hancox and Levi 1994b] and below). The heart was then pinned to a Sylgard dish and incisions made through the right ventricle and atrium. The Triangle of Koch was identified using established landmarks (Hancox et* *al. 1993) and the entire AVN region within this area was excised. Single cells were dispersed from the excised tissue through a combination of enzymatic and mechanical dispersion (Hancox et* *al. 1993). Cells were stored in Kraftbrühe (KB) solution (Isenberg and Klockner 1982; Hancox et* *al. 1993) at 4°C until use.

### Mouse AVN cell isolation

Mouse AVN cells were isolated using a modified method of the rabbit AVN cell isolation procedure. Male C57BL/6 mice (19–31 g) were killed humanely according to UK Home Office legislation. Mice received an intraperitoneal injection of Pentobarbital Sodium (Euthatal, 200 mg/mL, 0.24–0.48 mg/g of animal) and Heparin Sodium (25,000 I.U./mL, 30 I.U./g of animal). Following anesthesia, the heart was quickly removed and placed in a petri dish containing ice-cold solution comprising (in mmol/L): 130 NaCl, 4.5 KCl, 3.5 MgCl, 0.4 NaH_2_PO_4_, 0.75 CaCl_2_, 5 N-2-hydroxyethylpiperazine -N’-2-ethanesulfonic acid (HEPES), and 10 glucose, pH 7.25, plus 10 U/mL heparin. The heart was manually palpated to force the heparin-containing solution into the heart to remove blood and minimize the likelihood of clot formation. The dish containing the heart was then placed under a bench-top microscope and the aorta cannulated with a 21 gauge blunt needle attached to a 1 mL syringe filled with the same solution. The aorta was then tied onto the needle using a thin braided silk thread and solution was gently pushed into the heart through the aorta until solution ran clear (indicating removal of blood from the coronary circulation). The cannulated heart was then quickly mounted onto a Langendorff perfusion system and perfused consecutively at 37°C by three different oxygenated solutions (A–C), at a rate of 4 ± 0.5 mL/min. Solution A is the same solution as above, but with 1 mmol/L Ca^2+^ and was applied for 2 min. Solution B had the same composition as solution A, but omitted CaCl_2_ and included 0.1 mmol/L EGTA (ethylene glycol tetra-acetic acid) and was applied for 4 min. Solution C had the same composition as solution A, but with 0.24 mmol/L CaCl_2_, 1 mg/mL collagenase (Worthington, Type 1, CLSI) and 0.1 mg/mL protease (Sigma, Type XIV, Poole, Dorset, UK). This was applied for 6–9 min (visual inspection being used to monitor when the enzyme perfusate leaving the heart did so as a continuous “stream”, indicative of tissue digestion). Following the enzyme perfusion, the heart was removed and pinned down in a silicone-lined petri dish with the right atrium facing up. Surplus enzyme solution was washed away with KB solution (Hancox et* *al. 1993) and the heart was maintained in this KB solution throughout the dissection of the AVN under a microscope (Leica MZ6; magnification range 6.3–40×, generally used on low power). As for the AVN cell methods we previously developed for rabbit and guinea-pig (Hancox et* *al. 1993; Yuill and Hancox 2002), the AVN region was identified relative to anatomical landmarks (Marger et* *al. 2011b; Pauza et* *al. 2013) and the entire region encompassing the AVN and the posterior nodal extension, up to the coronary sinus (Pauza et* *al. 2013), was excised. The excised tissue was then gently mechanically agitated for 15 min at 37°C in enzyme-containing solution to which 17% bovine serum albumin (BSA) had been added. At the end of that period, the tissue was removed and placed into 1 mL of KB solution where it was manually dispersed with a very fine aperture fire polished glass pipette for 2–5 mins. The remaining tissue was mechanically agitated in enzyme for a further 10 min and cells then dispersed in KB; this was repeated twice (giving a total of three samples). The cells were kept in KB solution (Isenberg and Klockner 1982; Hancox et* *al. 1993) at 4°C and were used within 8 h.

### AVN single cell electrophysiology

Cells were placed in an experimental chamber mounted on the stage of an inverted microscope (Diaphot TMD or Eclipse TE2000-U, Nikon, Japan) and superfused with a Tyrode’s solution containing (in mmol/L) 140 NaCl, 4 KCl, 2 CaCl_2_, 1 MgCl_2_, 10 glucose, 5 HEPES [pH 7.4 with NaOH]). Whole-cell patch-clamp recordings were made using an Axopatch-1D amplifier, (Axon Instruments, now Molecular Devices, Sunnyvale, CA). Patch-pipettes (A-M Systems, Sequim, WA, USA) were pulled and heat-polished to a final resistance of 1.2–2.5 MΩ (Narishige PP-83 and Narishige MF-83, Japan). Protocols were generated and data recorded on-line with pClamp 9 or 10.0 software (Axon Instruments, USA) via an analog-to-digital converter Digidata 1200 or 1322 (Axon Instruments/Molecular Devices, USA). Data were digitized at 10 kHz with an appropriate bandwidth set on the recording amplifier. Data analysis was performed using Clampfit from the pClamp 9 and 10.0 software suite. Statistical analysis was performed using Microsoft Office Excel (Microsoft Corporation), Prism (Graphpad Software, Inc., La Jolla, CA, USA) and IgorPro (WaveMetrics, Lake Oswego, OR, USA). Data are presented as mean ± SEM. “*n*” values represent number of cells from which data were obtained and for all observations were derived from cells from at least two hearts.

The current–voltage (*I*–*V*) relation for L-type calcium current (*I*_Ca,L_) in Figure3 was fitted with the following equation:

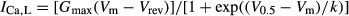
1where *G*_max_ is maximal *I*_Ca,L_ conductance, *V*_m_ is the test potential at which *I*_Ca,L_ was measured, *V*_rev_ is the reversal potential determined from extrapolation of the ascending limb of plotted current–voltage relations, *V*_0.5_ is the potential at which *I*_Ca,L_ activation is half maximal and *k* is the slope factor for current activation.

The *I*–*V* relations for (rapid) delayed rectifier tail currents in Figure4 (both those obtained in normal Tyrode’s solution and those obtained as E-4031-sensitive tail currents) were fitted with the following equation:

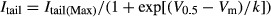
2where *I*_tail_ represents the tail current amplitude recorded at −40 mV following a given test pulse membrane potential (*V*_m_) and I_tail(Max)_ is the tail current with the greatest amplitude elicited by the protocol; *V*_0.5_ and *k* have similar meanings to those for equation 1.

### Recording solutions

The basic Tyrode’s solution described above was used for all experiments except measurements of sodium-calcium exchange current (*I*_NCX_) which utilized potassium-free Tyrode’s solution containing 10 μmol/L nitrendipine (to inhibit L-type calcium current) and 10 μmol/L strophanthidin (to inhibit the Na^+^/K^+^ pump) (Convery and Hancox 2000; Cheng et* *al. 2011). The patch-pipette solution for whole cell ionic current recording contained KCl 110, NaCl 10, HEPES 10, K_4_BAPTA 5, MgCl_2_ 0.4, Glucose 5, K_2_ATP 5, GTP–Tris 0.5, (pH 7.1 with KOH)(Choisy et* *al. 2012). For action potential (AP) measurements a similar patch solution, but omitting BAPTA, was used, with the addition of *β*-escin (50 μmol/L; [Marger et* *al. 2011b]) in the perforated-patch recording mode. For whole cell *I*_NCX_ recording, a Cs^+^-based internal solution was used that contained (in mmol/L): 110 CsCl, 10 NaCl, 0.4 MgCl_2_, 1 CaCl_2_, 5 EGTA, 10 HEPES, 5 glucose, 20 TEACl (pH 7.2 with CsOH) (Convery and Hancox 2000; Cheng et* *al. 2011). Once the whole-cell patch-clamp recording configuration had been obtained, cell superfusates were applied at 35–37°C via a home-built rapid solution exchange device (<1 s solution exchange time) (Levi et* *al. 1996). Drugs used were obtained from Sigma-Aldrich UK, with the exception of E-4031 which was generously donated by Eisai (Japan). E-4031 was dissolved in deionized water as a 10 mmol/L stock. Ryanodine was dissolved in methanol at 2.5 mmol/L. Nitrendipine was dissolved in methanol as a 10 mmol/L stock; strophanthidin was dissolved in ethanol as a 10 mmol/L stock. Aliquots of stock solution were added to external superfusate to give the final concentrations in the “Results” text. The % of vehicle in the final experimental solution, where stocks in organic solvents were used, was 0.1% or less.

## Results

### Cell capacitance, membrane resistance and currents activated by hyperpolarization

**Figure 1:**
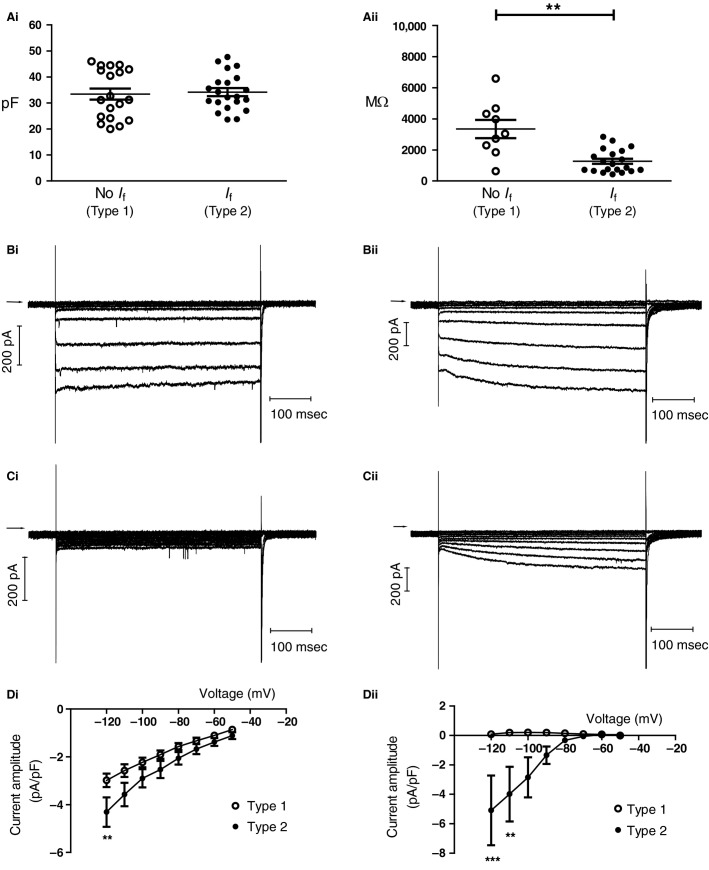
Membrane passive properties and responses to hyperpolarizing voltage steps of mouse atrioventricular nodal cells. (Ai and Aii) Plots of capacitance (pF) and input resistance (MΩ) of Type 1 and Type 2 cells. For Ai data from 19 Type 1 cells (open circles) and 21 Type 2 cells (filled circles) are shown. Thin horizontal line denotes mean and shorter lines show ±SEM. Aii uses a similar display format, showing data from 9 Type 1 cells and 20 Type 2 cells. Comparisons between Type 1 and 2 cells in Ai and Aii utilized an unpaired *t* test; in (Aii) a Welch-corrected test was used. (Bi and Bii) Representative ionic currents elicited from Type 1 (Bi) and Type 2 (Bii) cells elicited by 500 msec voltage commands from −40 mV to potentials between −40 and −120 mV (in 10 mV increments; start-to-start interval of 5 sec). (Ci and Cii) Representative ionic currents elicited from Type 1 (Ci) and Type 2 (Cii) cells (same cells as panel B) elicited by 500 msec voltage commands from −40 mV to potentials between −40 and −120 mV, in the presence of 1 mmol/L Ba^2+^. Horizontal arrows in B and C indicate zero current level. (Di and Dii) Mean *I*–*V* relationships in 1 mmol/L Ba^2+^ for (Di) instantaneous current on membrane potential hyperpolarization and (Dii) time-dependent (end-pulse minus start-pulse) current in Type 1 and Type 2 cells. For Di, *n* = 7 Type 1 cells and 6 Type 2 cells. For Dii, *n* = 6 Type 1 and 5 Type 2 cells. Asterisks denote statistical significance (2-way ANOVA with Bonferroni post hoc test; **P < 0.001, ***P < 0.0001).

Cell heterogeneity has been reported for cells isolated from the rabbit and guinea-pig AVN (Hancox and Levi 1994b; Munk et* *al. 1996; Ren et* *al. 2006). Early investigations of rabbit Ca^2+^ tolerant AVN cells showed that some cells exhibited a hyperpolarization-activated “funny” current, *I*_f_, whilst others did not (Hancox et* *al. 1993; Hancox and Levi 1994b); we termed the latter “Type 1” cells, and the former, “Type 2” cells (Hancox and Levi 1994b). We adopted a similar approach here in investigating the basic membrane properties of murine AVN cells and characterizing currents activated on membrane hyperpolarization. Membrane capacitance, which reflects cell surface area, was measured following capacitance compensation under whole cell voltage clamp. We have previously demonstrated close concordance between this method of capacitance measurement of AVN cells and calculation from capacitative current transients (Hancox et* *al. 1993). Figure1Ai shows plots of membrane capacitance values from Type 1 and 2 mouse AVN cells, with respective means of a 33.4 ± 2.2 pF (*n* = 19) and 34.2 ± 1.5 pF (*n* = 21; ns, *P* > 0.3 unpaired *t* test). Membrane resistance, measured under voltage clamp by applying small (±10 mV) voltage-excursions from −40 mV, was higher in Type 1 than Type 2 cells (Fig.1Aii), with mean values of 3344 ± 586 MΩ in Type 1 cells (*n* = 9) and 1267 ± 167 MΩ in Type 2 cells (*n* = 20), albeit with a wider range of values for Type 1 than Type 2 cells (*P* < 0.001 unpaired *t* test with Welch correction).

Figure1Bi and Bii show, for Type 1 and Type 2 cells, respectively, the inward currents activated by 500 msec duration hyperpolarizing voltage commands from −40 mV to a range of more negative voltages (10 mV increments). Initial steps in the protocol elicited small inward currents, but progressively larger voltage excursions elicited substantial inward currents. In Type 2 cells, however, a clear increase in current amplitude throughout the applied voltage command became evident, which was absent in Type 1 cells. In both cell types a large, rapid inward current occurred on repolarization to −40 mV (Fig.1Bi and Bii). Figure1Ci and Cii show currents from the same cells as Bi and Bii in the presence of 1mM Ba^2+^. In both Type 1 and Type 2 cells, Ba^2+^ blocked a substantial component of the current seen on membrane potential hyperpolarization. In Type 1 cells, hyperpolarizing pulses now elicited small, time-independent currents (Fig.1Ci), whilst a clear time-dependent hyperpolarization-activated current remained in Type 2 cells (Fig.1Cii). The large rapidly activating and inactivating inward current on repolarization to −40 mV from hyperpolarized potentials is clearly evident in the recordings from both cell types in the presence of Ba^2+^ (Fig.1Ci and Cii) and its size and rapid time-course are consistent with identity as rapid Na current (*I*_Na_) (Hancox et* *al. 1993; Hancox and Levi 1994b; Munk et* *al. 1996; Marger et* *al. 2011b). Figure1Di shows *I*–*V* relations for the instantaneous current in the presence of Ba^2+^ elicited immediately on membrane potential hyperpolarization for Type 1 (*n* = 7) and Type 2 (*n* = 6) cells, with inward currents that differed significantly only at −120 mV. Figure1Dii shows time-dependent current (the difference between end-pulse current and current immediately on membrane potential hyperpolarization) in the two cell types (Type 1, *n* = 6; Type 2, *n* = 5), in the presence of Ba^2+^ (Hancox and Levi 1994b). In Type 1 cells lacking *I*_f_, no significant time-dependent current was seen over the voltage range tested. In Type 2 cells, a clear time-dependent *I*_f_ was evident (see also Fig.1Cii); this increased in magnitude with progressively greater hyperpolarization. As is clear from the error bars for Type 2 cells in Figure1Dii, there was considerable cell-to-cell variation in the magnitude of this current in the cells studied so that, in statistical terms, significant differences from Type 1 cells were only seen between −110 and −120 mV.

**Figure 2:**
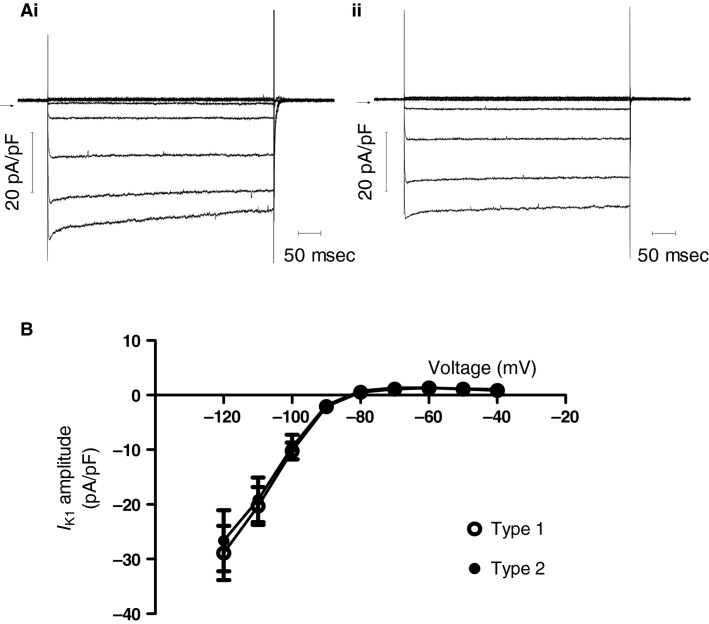
Inward rectifier K^+^ current. Horizontal arrow denotes zero current level. (B) *I*–*V* relation for Ba^2+^-sensitive *I*_K__1_ (measured at start of voltage command) in Type 1 (open circle; *n* = 7) and Type 2 (filled circle, *n* = 6) cells.

Figure2Ai and ii show the 1 mmol/L Ba^2+^-sensitive current for Type 1 and Type 2 cells. The Ba^2+^-sensitive current was small and outwardly directed at the holding potential of −40 mV and became inward and increasingly large at potentials negative to −80 mV. This is clearly shown in the *I*–*V* relation for Ba^2+^-sensitive current in Figure2B, which shows mean data superimposed for Type 1 and Type 2 cells. The mean *I*–*V* relations for both cell types were strongly inwardly rectifying and intersected the voltage axis at ∼−82 mV. These properties are consistent with an identity of the Ba^2+^-sensitive current as inwardly rectifying K^+^ current, *I*_K1_ (cf. [Yuill and Hancox 2002; Marger et* *al. 2011b]).

Comparison between the properties of currents from mouse and rabbit AVN cells may facilitate future adaptation/modification of existing rabbit AVN cell models (Inada et* *al. 2009) for mouse AVN. We therefore compared magnitude of time-dependent (end-pulse minus start-pulse) *I*_f_ in type 2 AVN cells of the two species, utilizing current measurements at −120 mV. For the five myocytes from which the data for *I*_f_, in the presence of Ba^2+^, were obtained (Fig.1Dii), the mean time-dependent *I*_f_ density was −5.1 ± 2.4 pA/pF. We also measured time-dependent (end-pulse minus start pulse) current in seven murine AVN cells in the absence of Ba^2+^, obtaining a mean density of −9.1 ± 2.1 pA/pF (*P* > 0.2; unpaired *t*-test). Due to the known sparsity of *I*_K1_ channels in rabbit nodal cells (Noma et* *al. 1984; Hancox et* *al. 1993), Ba^2+^ was not applied and *I*_f_ was measured as time-dependent (end-pulse minus start-pulse) hyperpolarization-activated current at −120 mV, as in previous studies (Hancox and Levi 1994b; Cheng et* *al. 2009; Choisy et* *al. 2012). The mean *I*_f_ density obtained was −1.5 ± 0.3 pA/pF (*n* = 18), which was significantly smaller than that in mouse cells (*P* < 0.05 vs. murine *I*_f_ with *I*_K1_ inhibition).

### *I*_Ca,L_

**Figure 3:**
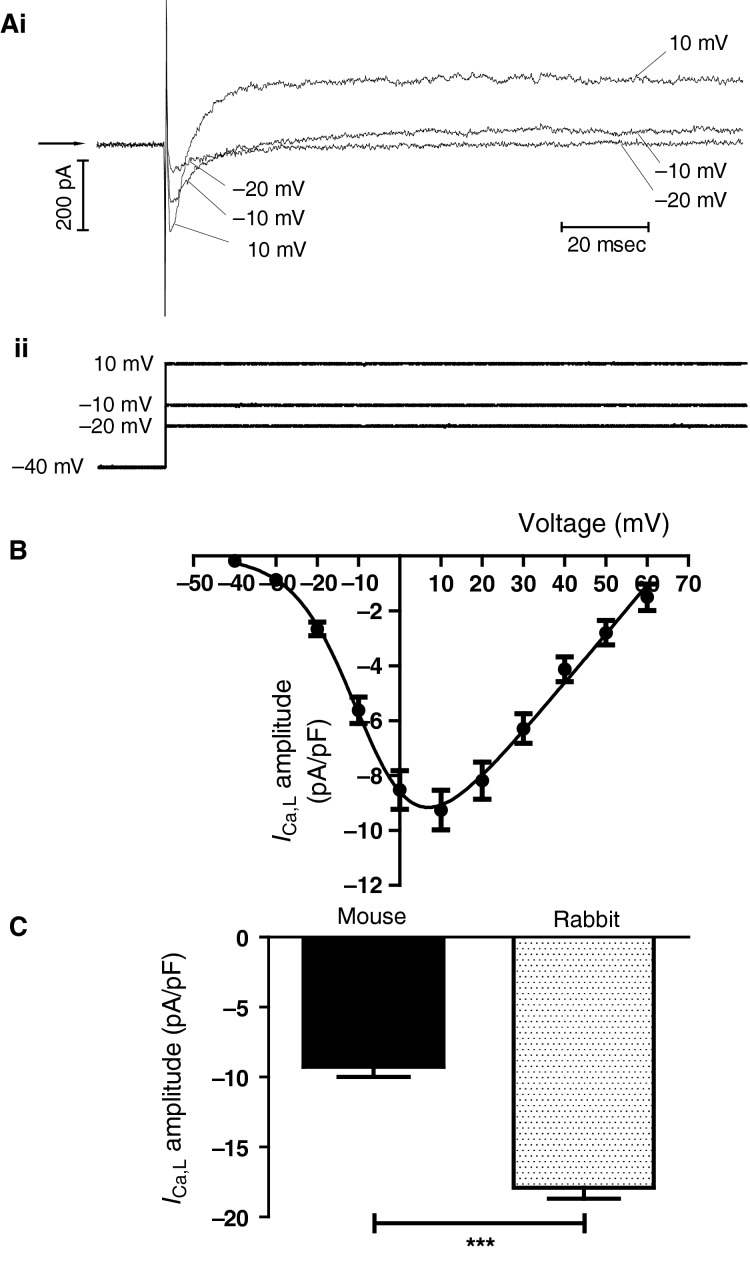
*I*_C__a,L_. (A) Representative records (upper traces, i) for *I*_C__a,L_ elicited at −20, −10 and +10 mV from a holding potential of −40 mV, by protocol shown in lower traces (ii). (B) *I*–*V* relation for *I*_C__a,L_ (*n* = 24). Data were fitted with equation 1 in the Materials and Methods, to give a *V*_0.5_ of −7.6 ± 1.2 mV and a slope factor (*k*) of 7.6 ± 0.9 mV. Note that there was no significant difference of *I*_C__a,L_ amplitude between cells containing or lacking *I*_f_ (see Results text for peak density at +10 mV comparison) and so data were pooled. (C) Comparison of current density (pA/pF) of *I*_C__a,L_ at +10 mV in mouse (*n* = 24) and rabbit (*n* = 29) AVN cells. Asterisks denote statistical significance at ***P < 0.0001 (unpaired *t*-test).

*I*_Ca,L_ plays an important role in AP generation and conduction in the AVN in other species (Zipes and Mendez 1973; Zipes and Fischer 1974; Hancox et* *al. 2003) and is also important to electrogenesis of mouse AVN cells (Marger et* *al. 2011a,b; Zhang et* *al. 2011). Here we measured *I*_Ca,L_ from mouse AVN cells under “physiological” recording conditions (with a K^+^-based pipette dialysate and standard Tyrode’s solution at physiological temperature) under conditions similar to those used in prior rabbit AVN cells studies (e.g. [Cheng et* *al. 2009; Choisy et* *al. 2012]). 500 msec duration voltage commands were applied in 10 mV increments from a holding potential of −40 mV (to inactivate fast Na channels [Hancox et* *al. 1993; Hancox and Levi 1994a,b]). *I*_Ca,L_ was measured as the peak of the early inward current observed on depolarization. Figure3A shows representative traces of currents elicited by this protocol at −20, −10 and +10 mV; *I*_Ca,L_ activated rapidly, reaching a peak within 2–4 msec of onset of the depolarizing voltage command. The peak of the current was small at −20 mV and increased at test voltages of −10 and +10 mV and, at the latter potential, gave way to a substantial outward current with maintained membrane potential depolarization. Figure3B shows the mean *I*–*V* relation for peak *I*_Ca,L_ (normalized to cell capacitance) for 24 cells. The mean data were fitted with equation 1 (see Materials and Methods), to derive parameters describing *I*_Ca,L_ activation. The *V*_0.5_ for *I*_Ca,L_ activation was −7.6 ± 1.2 mV with a slope factor (*k*) of 7.6 ± 0.9 mV. Current was maximal at +10 mV, whilst maximal macroscopic conductance (*G*_max_) estimated from the fitted data was 0.18 nS/pF. When cells were sub-grouped according to the presence/absence of *I*_f_, there was no significant difference in peak *I*_Ca,L_ amplitude between Type 1 (−9.2 ± 0.7 pA/pF; *n* = 15) and Type 2 (−9.4 ± 1.5 pA/pF; *n* = 9) cells. Figure3C compares the amplitude of peak *I*_Ca,L_ at +10 mV in mouse AVN cells with that from rabbit AVN cells under the same recording conditions; rabbit AVN *I*_Ca,L_ was significantly larger than that in mouse.

### *I*_Kr_

**Figure 4:**
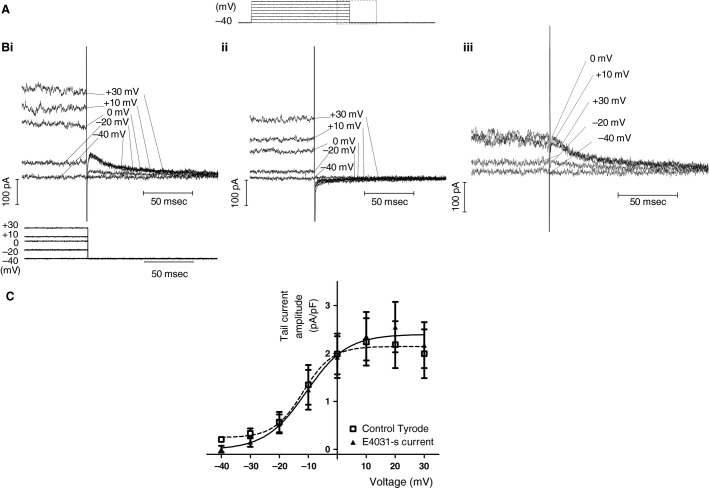
E-4031-sensitive *I*_K__r_. (A) Schematic representation of square pulse protocol used (applied between −40 mV and +30 mV in 10 mV increments (start to start interval of 5 sec). The boxed area represents the corresponding portion of ionic currents displayed in (B), in order to optimise display of tail currents. (B) Selected currents at −40, −20, 0, +10 and +30 mV in normal Tyrode’s solution (Bi), in 5 μmol/L E-4031 (Bii) and as E-4031-sensitive current (Biii). Corresponding portion of voltage protocol is shown as lower traces in (Bi). Note that current axis in (Biii) differs slightly from that in (Bi) and (Bii). (C) *I*–*V* relations for tail currents obtained in control Tyrode solution (filled square, *n* = 8) and for E4031-s current (filled triangle, *n* = 8). Data for Type 1 and 2 cells were pooled. The plots were fitted with equation 2 (Materials and Methods), to give *V*_0.5_ and *k* values in normal Tyrode of −11.8 ± 4.3 mV (*V*_0.5_) and 5 ± 3.9 mV; for E-4031-sensitive current, the corresponding values were −10.7 ± 4.7 mV (*V*_0.5_) and 7 ± 4.4 mV.

Delayed rectifier K^+^ current can be measured reliably from rabbit, guinea-pig and rat AVN cells from outward “tail” currents that occur on repolarization after depolarizing voltage-clamp commands (Howarth et* *al., 1996; Habuchi et* *al. 1995; Mitcheson and Hancox 1999; Yuill and Hancox 2002; Cheng et* *al. 2009; Yuill et* *al. 2010). We adopted a similar approach to evaluate delayed rectifier current in mouse AVN cells. Figure4A shows a schematic representation of the protocol used to elicit the current: 500 msec depolarizing pulses between −40 and +30 mV, with 10 mV increments in successive applications, were applied in normal Tyrode’s solution. Figure4Bi shows representative current records at selected potentials, expanded to display the tail current observed on repolarization to −40 mV. Tail current amplitude increased progressively following successively larger depolarizing commands until ∼ 0 to +10 mV, and then did not increase with further depolarization. Figure4Bii shows currents from the same cell after exposure to a supramaximal concentration (5 μmol/L) of the *I*_Kr_ inhibitor E-4031, which reduced current amplitude during the depolarizing pulse and abolished outward tail currents. Figure4Biii shows E-4031-sensitive currents (i.e., *I*_Kr_) from the same experiment, showing overlap of tail currents at 0 mV and above. Figure4C shows that the mean *I*–*V* relations for tail currents in normal Tyrode’s solution and for E-4031-sensitive currents were similar. Fitting the data-sets with equation 2 (see Materials and Methods) yielded *V*_0.5_ and *k* values for the net tail current of –11.8 ± 4.3 mV (*V*_0.5_) and 5 ± 3.9 mV (*k*) and for the E-4031-sensitive current of −10.7 ± 4.7mV (*V*_0.5_) and 7 ± 4.4 mV (*k*). Both the lack of residual outward tail current in the presence of *I*_Kr_ inhibition (Fig.4Bii) and the close concordance between net tail current and E-4031-sensitive tail current *I*–*V* relations (Fig.4C) indicate current identity as *I*_Kr_ and that the slow delayed rectifier K^+^ current (*I*_Ks_) was functionally absent in murine isolated AVN cells under these recording conditions. There was a trend toward a greater *I*_Kr_ amplitude (3.3 ± 0.6 pA/pF; *n* = 5) in *I*_f_ containing Type 2 cells than in Type 1 cells from which *I*_f_ was absent (1.3 ± 0.1 pA/pF; *n* = 3) following the test pulse to +20 mV, *P* < 0.05 (unpaired *t*-test Welch correction).

**Figure 5:**
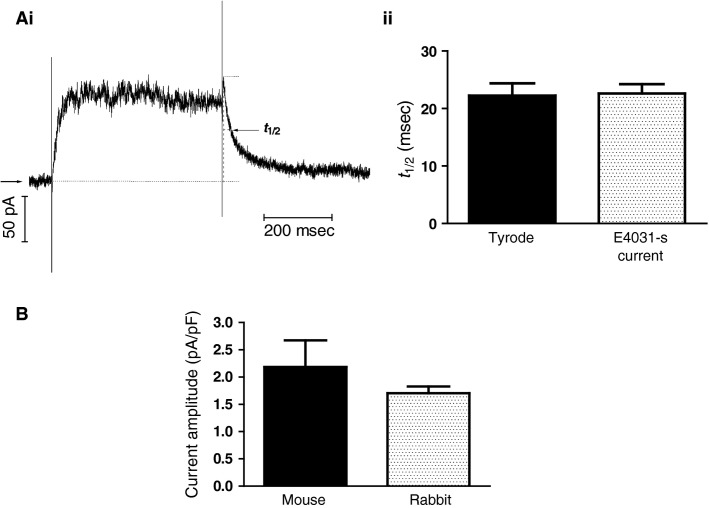
*I*_K__r_ deactivation. (Ai) Representative trace for E4031-sensitive current by 500 msec voltage clamp command to +20 mV. *t*_1/2_ (msec) indicates the time for the “tail” current to decrease to 50% of its maximal amplitude as indicated by the dotted lines on the trace. (Aii) Mean *t*_1/2_ values for tail currents in normal Tyrode’s solution (*n* = 12) and E4031-s (*n* = 12). Data come from repeated application of single commands to +20 mV. (B) Comparison of “tail” current density at −40 mV following commands to +20 mV, in cells from mouse (*n* = 8) and rabbit (*n* = 23) AVN. Data from Type 1 and Type 2 cells were pooled.

We performed additional characterization of *I*_Kr_ by quantifying the time-course of tail current deactivation. This was achieved by measuring the *t*_1/2_ of deactivation for both net tail current and E-4031-sensitive current. Figure5Ai indicates how *t*_1/2_ was measured, for an E-4031-sensitive current elicited by a test command to +20 mV. Figure5Aii shows mean *t*_1/2_ data for tail current deactivation for net current and for E-4031-sensitive current (NSD between the two). Finally, we compared the peak tail current density at -40 mV following depolarization to +20 mV between mouse (*n* = 8) and rabbit (*n* = 23) AVN cells. There was no significant difference between the two (*P* > 0.05).

### *I*_NCX_

**Figure 6:**
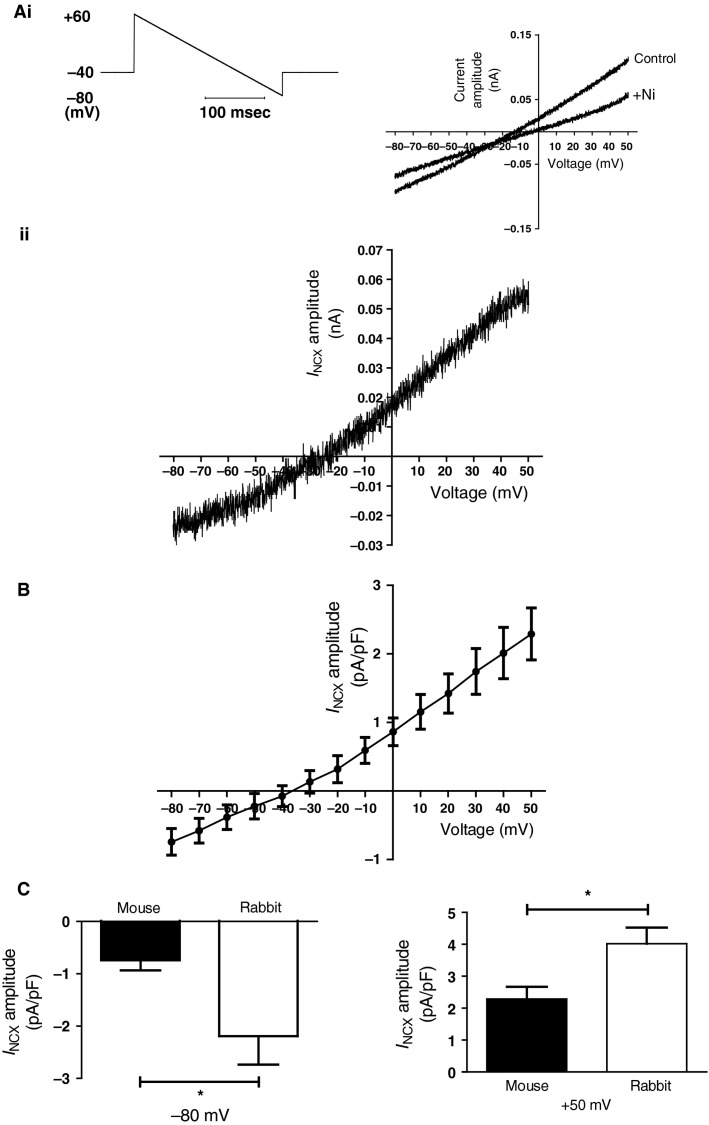
*I*_N__aCa_.(Ai) Representative descending voltage ramp protocol (left hand side) used to elicit *I*_N__aCa_, and current traces elicited by this protocol (right hand side) in absence (control) and presence of 5 mmol/L Ni^2+^ (+ Ni). Start to start interval of the protocol was 3 sec. (Aii) Representative current trace of the Ni^2+^-sensitive current: *I*_N__aCa_ (obtained by subtracting the residual current recorded in the presence of Ni^2+^ from that in control. (B) *I*–*V* relationship for Ni^2+^-sensitive *I*_N__aCa_ (*n* = 6). Ni^2+^-sensitive currents were sampled at 10 mV intervals, normalized to current density and pooled. (C) Comparison of the *I*_N__aCa_ current amplitude measured in mouse (*n* = 6) and in rabbit (*n* = 5) atrioventricular nodal cells at −80 and +50 mV. Asterisks denote statistical significance (unpaired *t* test; *P* < 0.05).

Prior data from rabbit AVN cells provide strong evidence for the presence and functional importance of electrogenic Na-Ca exchange current, *I*_NCX_ (Hancox et* *al. 1994; Convery and Hancox 2000; Ridley et* *al. 2008; Cheng et* *al. 2011) in this cell type. To our knowledge, however, *I*_NCX_ from mouse AVN cells has not hitherto been studied. Organic Na-Ca inhibitors generally show poor selectivity (Doggrell and Hancox 2003). Consequently, we undertook *I*_NCX_ measurements from murine AVN cells using conditions similar to those used in prior rabbit AVN cell studies (Convery and Hancox 2000; Cheng et* *al. 2011). Pipette and superfusate solutions were used that eliminated major overlapping time and voltage-dependent conductances; under these conditions *I*_NCX_ can be measured as current sensitive to 5 mmol/L Ni^2+^ (Convery and Hancox 2000; Cheng et* *al. 2011). Figure6A shows the voltage-ramp protocol used (Fig.6Ai), together with representative traces in the absence and presence of Ni^2+^. Figure6Aii shows the resulting Ni-sensitive current from the same experiment, whilst Figure6B shows mean *I*–*V* data from six experiments. To construct this plot, Ni^2+^-sensitive current densities at 10 mV intervals during the voltage-ramp were pooled. The weakly outwardly rectifying *I*–*V* relation, with inward current at voltages negative to −40 mV is similar to that previously recorded from rabbit AVN cells (Convery and Hancox 2000; Cheng et* *al. 2011). The *I*_NCX_-selective conditions used in these experiments precluded identification of cells as Type 1 or Type 2 and so comparison between the two cell types was not possible. However, we were able to compare the magnitude of mouse AVN *I*_NCX_ with that from rabbit AVN cells at both positive (+50 mV) and negative (−80 mV) voltages. As shown in Figure6C, although a robust *I*_NCX_ was present in mouse AVN cells, it was significantly smaller than that from rabbit AVN cells recorded under identical conditions.

### Spontaneous APs and effect of ryanodine

**Figure 7:**
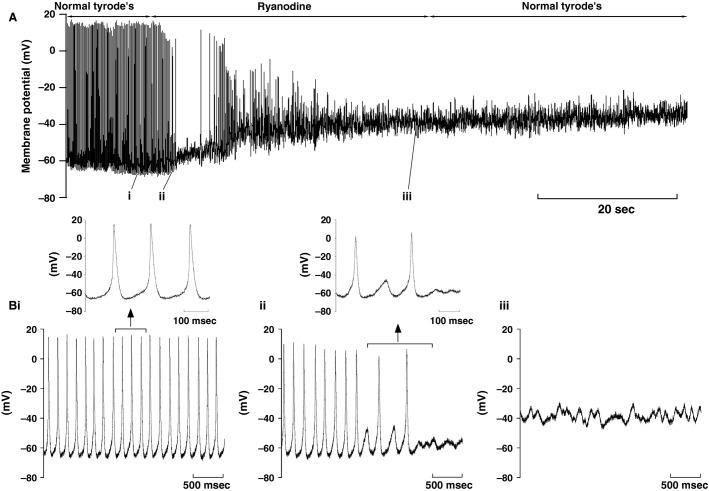
Effects of ryanodine on spontaneous APs. (A) Slow time-base recording of APs before, during and after the application of 1 μmol/L ryanodine. (B) Expanded (faster time-base) recordings from the numbered sections of panel A (i, ii and iii). The inserts above (Bi) and (Bii) show APs on an expanded time-scale. Similar results were observed in 4 cells.

In a final series of experiments we made AP recordings from spontaneously active AVN cells. It transpired to be more difficult to make AP recordings than recordings of ionic currents. Many cells did not survive “current clamp” recording mode for more than a few seconds and we observed that with whole-cell patch-clamp recording and associated intracellular dialysis with the pipette solution, spontaneous activity (evident visually as regular spontaneous cell beating) was usually lost on gaining the whole-cell recording mode. This suggests that spontaneous activity was sensitive to intracellular dialysis. Marger and colleagues reported successful murine AVN AP measurements using escin-perforated patch (Marger et* *al. 2011b) and therefore we also applied this method. AP recordings were still difficult to achieve, although we were able to obtain viable recordings in six experiments with this method. However, it was not possible in these experiments to perform voltage-clamp measurements at the end of AP recording to ascertain whether the cells studied were “Type 1” or “Type 2” in respect of absence or presence of *I*_f_. Figure7A shows results from one of these experiments. The main panel shows a comparatively slow time-base recording, whilst faster time-base extracts are shown as insets. In normal Tyrode’s solution, the following mean AP parameters were obtained: a spontaneous AP rate of 5.2 ± 0.5 sec^−1^; a maximum diastolic potential of −64 ± 4.8 mV; a diastolic depolarization rate 0.221 ± 0.059 V/s, an AP upstroke velocity of 37.7 ± 16.2 V/s, peak overshoot potential of 30.8 ± 6.7 mV; duration at 50% repolarization of 18.2 ± 3.5 msec (*n* = 6). Spontaneous activity of rabbit AVN cells has been shown to be sensitive to inhibitors of sarcoplasmic reticulum (SR) Ca^2+^ release and re-uptake (Ridley et* *al. 2008; Cheng et* *al. 2011). We were able to apply 1 μmol/L ryanodine to 4 cells. As shown in Figure7 (Fig.7A and Bi in normal Tyrode’s solution, Figure7Bii in ryanodine and Figure7Biii following return to normal Tyrode’s solution), ryanodine exposure led to irreversible loss of spontaneous APs, which gave way to small subthreshold membrane potential oscillations and quiescence. Similar results were seen in each of the cells studied.

## Discussion

### Basic properties of mouse AVN cells in context

The AVN is structurally and electrically heterogeneous (Meijler and Janse 1988; Munk et* *al. 1996; Hancox et* *al. 2003; Efimov et* *al. 2004; Ren et* *al. 2006) and it is not possible to ascertain the origin from within the AVN region of individual isolated cells. Histological examination of the mouse AVN showed a transitional region as well as compact node and posterior nodal extension (Nikmaram et* *al. 2008; Marger et* *al. 2011b). Whilst acknowledging heterogeneity in the isolated cell population, Marger and colleagues reported characteristics only of spontaneously active, *I*_f_-possessing cells in their electrophysiological characterization of murine AVN cells (Marger et* *al. 2011a,b). Here we adopted an alternative, previously validated practice of grouping cells using a method that does not attempt to assign origin from within the AVN region, but instead categorises cells according to the presence/absence of *I*_f_ (Hancox and Levi 1994b). There was no difference in mean cell surface area (as measured by cell capacitance) between Type 1 and Type 2 (*I*_f_ possessing) AVN cells in this study, though the mean cell capacitance of *I*_f_-possessing cells in our study (34 pF) is higher than that reported by Marger and colleagues (20 pF) (Marger et* *al. 2011b). The reason for this difference is unclear, but it may reflect differences between the two studies in cell isolation protocols and enzymes, which could differentially influence cell dispersion and survival. However, the capacitance values reported here are within the range of mean values of between 25 and 41 pF (Munk et* *al. 1996; Yuill and Hancox 2002; Hancox et* *al. 2003; Yuill et* *al. 2010) reported previously for rat, rabbit and guinea-pig AVN cells. The lability of membrane potential of pacemaker cells has long been recognized to correlate with high values of membrane resistance, which mean that small currents can produce significant membrane potential changes (Irisawa et* *al. 1993; Hancox et* *al. 2003). Membrane resistance values ranging between ∼565 MΩ and 1.2 GΩ have been reported for morphologically normal rabbit AVN cells (Hancox et* *al. 1993; Martynyuk et* *al. 1995; Munk et* *al. 1996) and of ∼1.1 GΩ and ∼1.4 GΩ for rat and guinea-pig AVN cells respectively (Yuill and Hancox 2002; Yuill et* *al. 2010). The high membrane resistance in guinea-pig AVN cells is striking as this occurs despite the presence of an *I*_K1_; however, this current is only of significant amplitude at potentials negative to the diastolic range (Yuill and Hancox 2002). Murine AVN cells appear to resemble guinea-pig AVN cells in this respect: ∼1.3–3.3 GΩ membrane resistance values were obtained in the present study despite the presence of a Ba^2+^-sensitive *I*_K1_ in both Type 1 and Type 2 cells. However, in both cell groups *I*_K1_ was very strongly rectifying, with substantial currents occurring only at potentials negative to the observed pacemaker potential range. Mangoni et* *al. also observed a Ba^2+^-sensitive *I*_K1_ in AVN cells, routinely using 5 mmol/L Ba^2+^ in their *I*_f_ recordings (Marger et* *al. 2011b). The functional relevance of *I*_K1_ current in the AVN of any species remains to be elucidated.

The mean spontaneous AP rate of mouse isolated AVN cells in the present study (5.2 sec^−1^) is faster than that reported by Marger and colleagues (2.9 sec^−1^) (Marger et* *al. 2011a,b). The maximal upstroke rate of AVN cell spontaneous APs reported by Marger et* *al. (13 V/s) was also much slower than that seen in the present study (37.7 V/s). On the other hand, our values are comparable to those reported previously for murine isolated SAN cells by other workers (Lei et* *al. 2004, 2005). Thus, a spontaneous cycle length of 157 msec (equivalent to an AP rate of ∼6.4 sec^−1^), maximal upstroke velocity of 48 V/s and overshoot potential of 35 mV reported for mouse SAN cells (Lei et* *al. 2004) are all similar to the mean values seen here. Rabbit AVN cell upstroke velocities are considerably slower (Hancox et* *al. 1993; Munk et* *al. 1996) than those seen here for mouse AVN cells. The fast upstroke velocity of murine AVN APs is consistent with a significant role for *I*_Na_ in AVN AP electrogenesis in mouse, whereas by contrast Na^+^ channels are sparse within the compact node region of the rabbit AVN (Petrecca et* *al. 1997). Consistent with this, Marger et* *al. found spontaneous APs in mouse AVN cells to be sensitive to micromolar concentrations of tetrodotoxin (TTX) (Marger et* *al. 2011b), whilst Nikmaram and colleagues reported a prolongation of AVN cycle length by TTX in mouse intact AVN tissue preparations (Nikmaram et* *al. 2008). Detailed characterization of *I*_Na_ was beyond the intended scope of the present study, but the fast upstroke velocity of AVN cell APs seen here suggests that future work to characterise further the biophysical properties of murine AVN cell *I*_Na_ (cf. [Marger et* *al. 2011b]) and the underlying Na channel isoform(s) (cf. [Lei et* *al. 2004]), would be useful.

### Hyperpolarization-activated current

Previous immunohistochemical work has shown strong staining of the mouse AVN region for the HCN4 channel isoform that significantly underpins pacemaker cell *I*_f_ (Nikmaram et* *al. 2008; Marger et* *al. 2011a,b). A partial (∼32%) blocking concentration of the *I*_f_ inhibitor ZD-7228 was reported to inhibit AVN cell spontaneous rate by 16% (Marger et* *al. 2011b), whilst cells from mice expressing a dominant negative cAMP-insensitive HCN4 isoform exhibited irregular pacemaking (Marger et* *al. 2011a). The *I*_f_ recorded from Type 2 AVN cells in this study was similar to that measured by Marger and colleagues (Marger et* *al. 2011b), although is a little smaller in amplitude. In part this might result from the inclusion of Ca^2+^chelator in our pipette solution, as *I*_f_ is sensitive to internal [Ca^2+^] (Hagiwara and Irisawa 1989). *I*_f_ was relatively small in our experiments over the diastolic potential range, which could also be influenced by low intracellular [Ca^2+^] (Hagiwara and Irisawa 1989). However, Marger et* *al. reported a smaller current density and more negative activation *V*_0.5_ for *I*_f_ in mouse AVN cells than SAN cells in a direct comparison (with respective *V*_0.5_ values of −111 and −101 mV), suggesting that *I*_f_ is more prominent over the diastolic potential range in mouse SAN than AVN. Interestingly, whilst expression of dominant negative, cAMP-insensitive HCN4-affected baseline pacemaking of mouse AVN cells, it did not impair the response to isoprenaline (Marger et* *al. 2011a). Thus, *I*_f_ appears to contribute to baseline pacemaker properties of murine AVN cells, but not to be a critical determinant of the response to *β*-adrenergic stimulation (Marger et* *al. 2011a). Our comparison of *I*_f_ amplitude at −120 mV suggests that *I*_f_ density is higher in mouse than in rabbit AVN cells. To make comparisons of voltage-dependence of *I*_f_ from the two species, in additional analysis (data not shown), we compared *I*–*V* relations for *I*_f_ in rabbit and mouse AVN cells, by normalizing currents for each species at each voltage to the current at −120 mV (normalized Ba^2+^-insensitive *I*_f_ data from the present study were compared with normalized rabbit *I*_f_ data from Choisy et* *al. 2012). This analysis method eliminated any difference in current density between AVN cells from the two species. The normalized data-plots were closely superimposable (no significant differences in currents between −40 and −120 mV except at −90 mV; 2-way ANOVA with Bonferroni post-test). Thus, the voltage dependence of *I*_f_ in the present study was similar to that observed in prior rabbit AVN cell experiments from our laboratory (Cheng et* *al. 2009; Choisy et* *al. 2012), over physiologically relevant voltages.

The Ba^2+^-insensitive instantaneous current on membrane potential hyperpolarization (Fig.1Di) did not differ significantly between Type 1 and 2 cells except at the negative extreme of the voltage-range tested, so is unlikely largely to be attributable to instantaneous current through *I*_f_ channels (Proenza et* *al. 2002), as Type 1 cells lack *I*_f_. Potential contributors to a distinct instantaneous current include Na-Ca exchange, which our experiments under Na-Ca exchange selective conditions (Fig.6) demonstrated to be present in these cells, and the Na-dependent background current (*I*_B,Na_), which has been observed in SAN cells (Hagiwara et* *al. 1992). The molecular basis of channels underpinning *I*_B,Na_ is not yet established and data on *I*_B,Na_ in cells from the AVN are currently lacking. The question as to whether or not *I*_B,Na_ could contribute to instantaneous inward current at negative voltages in AVN cells therefore merits dedicated investigation.

### *I*_Ca,L_

Both T-type (Ca_v_ 3.1; *I*_Ca,T_) and L-type (Ca_v_ 1.2 and 1.3) Ca^2+^ currents have previously been reported in mouse AVN cells (Marger et* *al. 2011a,b; Zhang et* *al. 2011). *I*_CaT_ has not been reported for morphologically normal AVN cells from rabbit hearts and biophysically detailed rabbit AVN cell models lack an *I*_CaT_ (Hancox et* *al. 2003; Inada et* *al. 2009). The present investigation aimed to study murine *I*_Ca,L_ under conditions similar to those used to investigate rabbit *I*_Ca,L_ (Hancox and Levi 1994a; Cheng et* *al. 2009; Choisy et* *al. 2012) and the holding potential used (−40 mV) would have inactivated mouse AVN cell *I*_Ca,T_. Marger and colleagues showed abolition of spontaneous activity by the dihydropyridine isradipine and separated *I*_Ca,T_ from *I*_Ca,L_ biophysically, using holding potentials of −90 mV and −55 mV to record total *I*_Ca_ and *I*_Ca,L_ respectively, thus obtaining *I*_Ca,T_ as the difference current (Marger et* *al. 2011b). The *V*_0.5_ of *I*_Ca,T_ in their study was −45 mV whilst that for *I*_Ca,L_ was −22 mV. Under their conditions, *I*_Ca,T_ was larger than was *I*_Ca,L_ (Marger et* *al. 2011b). In further experiments, the same authors found marked AVN dysfunction in Ca_v_ 1.3 and Ca_v_ 3.1 knockout mice (Marger et* *al. 2011a), results which suggested that both Ca_v_ 1.3 and 3.1 are important for spontaneous activity. Moreover, they found that whilst the presence of functional HCN4 channels was not obligatory for the chronotropic response to isoprenaline, Ca_v_ 3.1 channels were important for isoprenaline-induced rate acceleration (Marger et* *al. 2011a). An independent study by Zhang et* *al. used Ca_v_ 1.3 null mice to show a decrease in AP firing rate from the intact AVN in the absence of Ca_v_ 1.3 and presented characteristics of *I*_Ca,L_ in wild-type and Ca_v_ 1.3 null mice (Zhang et* *al. 2011). Using a similar holding potential of −55 mV to inactivate *I*_Ca,T_, they obtained *V*_0.5_ values of −11.8 mV in the presence of Ca_v_ 1.3 and −5.2 mV (reflecting properties of Ca_v_ 1,2) in its absence (Zhang et* *al. 2011). That the holding potential used in the present study would have inactivated *I*_Ca,T_ (as well as *I*_Na_) and would favour Ca_v_ 1.2 mediated *I*_Ca,L_ over that carried by Ca_v_ 1.3 is reflected in the *V*_0.5_ of *I*_Ca,L_ of −7.6 mV in our experiments, which lies between the values in cells from Ca_v_ 1.3 containing and Ca_v_ 1.3 null mice in the study of Zhang et* *al. (Zhang et* *al. 2011), but is closer to the latter. Under similar recording conditions in this study, we have previously obtained *V*_0.5_ values between −13.8 and −2.7 mV for rabbit AVN cell *I*_Ca,L_ (Cheng et* *al. 2009; Choisy et* *al. 2012), with *V*_0.5_ values of −18.2 and −0.5 mV reported, respectively, for rat and guinea-pig AVN cells under comparable conditions (Yuill and Hancox 2002; Yuill et* *al. 2010). The AVN cell maximal *I*_Ca,L_ magnitude in the present study (Fig.3B) was greater than that reported in the studies of either Marger et* *al. or Zhang et* *al. (Marger et* *al. 2011a,b; Zhang et* *al. 2011), with no significant difference between Type 1 and 2 cells. However, it was lower than that from rabbit AVN cells under similar conditions (Fig.3C; though differences between studies in rabbit AVN cell *I*_Ca,L_ density have previously been noted by Inada et* *al. [Inada et* *al. 2009]). It is also worth noting that the peak *I*_Ca,L_ density for mouse AVN cells in the present study is similar to that reported previously for guinea pig and rat AVN cells (Yuill and Hancox 2002; Yuill et* *al. 2010).

### Rapid delayed rectifier K^+^ current, *I*_Kr_

*I*_Kr_ is recognized to play a major role in AP repolarization and in influencing the diastolic depolarization of both SAN and AVN cells from the rabbit (Shibasaki 1987; Habuchi et* *al. 1995; Ono and Ito 1995; Howarth et* *al., 1996b;Mitcheson and Hancox 1999; Zaza et* *al. 1997; Sato et* *al. 2000; Inada et* *al. 2009). One of the notable differences in the cellular electrophysiology of rabbit SAN and AVN is the presence of both *I*_Kr_ and *I*_Ks_ in rabbit SAN cells (Habuchi et* *al. 1995; Lei et* *al. 2002), but absence of *I*_Ks_ in the AVN. The limited data on rat AVN delayed rectifier current, *I*_K_, also show activation parameters consistent with current identity as *I*_Kr_ with little or no contribution of *I*_Ks_ (Yuill and Hancox 2002). By contrast, the voltage-dependence of guinea-pig AVN cell *I*_K_ “tails” was best-described by a double-Boltzmann relationship, with *V*_0.5_ values for the two components of ∼ −17 mV and +27 mV, consistent with the presence of functional *I*_Kr_ and *I*_Ks_ in the AVN from that species.

To our knowledge, the present study is only the second to have measured murine AVN cell *I*_Kr_ (Marger et* *al. 2011b) and it is the first to quantify voltage-dependent activation of the current. Similar to the present study, Marger and colleagues reported ubiquitous presence of an E-4031-sensitive K^+^ current, without any detectable *I*_Ks_. Marger and colleagues found mouse AVN *I*_Kr_ to be larger than that in mouse SAN cells; the *I*_Kr_ density at positive voltages in their study is also greater than that for the pooled Type 1 and Type 2 cell data shown in Figure4 here, though is more similar to that of the Type 2 cells in our sample. The *V*_0.5_ for E-4031-sensitive *I*_Kr_ in the present study (∼−11 mV; Fig.4), is somewhat more positive than that reported for mouse SAN *I*_Kr_ (∼−24 mV), but matches closely that previously reported for rabbit AVN E-4031-sensitive *I*_Kr_ (∼−11 mV; (Mitcheson and Hancox 1999). The deactivation time-course of the current (Fig.5) is consistent with a maintained, deactivating *I*_Kr_ component during diastolic depolarization (cf. [Ono and Ito 1995; Mitcheson and Hancox 1999; Zaza et* *al. 1997]), in agreement with a reported effect of E-4031 on spontaneous cycle length of the intact mouse AVN (Nikmaram et* *al. 2008). Whilst the lack of residual outward tail currents in the presence of E-4031 in the present study (Fig.4) and the prior findings of Marger and colleagues both indicate that a functional *I*_Ks_ is absent from mouse AVN cells under baseline conditions, we do not exclude entirely the possibility that the current might be activated in some circumstances (for example, in the presence of *β*-adrenergic agonist, cf. [Lei et* *al. 2002]) and this warrants future experimental investigation.

### *I*_NCX_ and the effect of ryanodine

The pacemaker activity of the sinoatrial node (SAN) is now generally accepted to involve both sarcolemmal ion channels and intracellular calcium cycling with SR Ca^2+^ release coupled to electrogenesis via the Na-Ca^2+^ exchange (Sanders et* *al. 2006; Lakatta et* *al. 2010). Data from experiments on intact hearts and AVN preparations from both small and large model species, in which ryanodine and thapsigargin were used to inhibit SR function also support a role for SR Ca^2+^ release in influencing AVN pacemaking (Nikmaram et* *al. 2008; Kim et* *al. 2010; Cheng et* *al. 2012). Notably, mouse AVN spontaneous cycle length in an intact tissue preparation was increased by ∼240% by exposure to 2 μmol/L ryanodine (compared to 70% for the mouse SAN and 30% for the rabbit AVN in the same study [Nikmaram et* *al. 2008]). AVN single cell experiments on the rabbit AVN have shown that ryanodine and thapsigargin can induce quiescence in spontaneously active cells and have also implicated *I*_NCX_ in AVN pacemaking (Hancox et* *al. 1994; Ridley et* *al. 2008; Cheng et* *al. 2011). The present study is the first both in which direct measurements of Ni^2+^-sensitive *I*_NCX_ have been made from murine AVN cells and the first in which the AVN cell response to ryanodine has been examined. Our experiments yielded an *I*_NCX_ that is qualitatively similar to that reported previously for rabbit AVN cells (Convery and Hancox 2000; Cheng et* *al. 2011) though it is smaller in amplitude (Fig.6). The data in Figure7 with 1 μmol/L ryanodine showed a strong sensitivity of spontaneous APs in mouse AVN cells to SR Ca^2+^ release, which is consistent with prior observations from rabbit AVN cells (Ridley et* *al. 2008; Cheng et* *al. 2011). Indeed, the cessation of murine AVN cell spontaneous APs with ryanodine in the present study matches closely that seen previously in rabbit AVN cell experiments where exposure to 1 μmol/L ryanodine arrested spontaneous APs within ∼21 sec (Ridley et* *al. 2008; Cheng et* *al. 2011). Thus, our findings demonstrate that, in addition to previously identified roles in murine AVN cell spontaneous activity for *I*_Ca,L_, *I*_Ca,T_ and *I*_f_, SR Ca^2+^ release also plays an important role in influencing electrogenesis.

### Limitations, implications and conclusions

The mouse AVN cell isolation procedure used here was adapted from our rabbit AVN cell isolation procedure. For both species the process involves heart exposure to heparin (to prevent clot formulation), but only mice received pentobarbital. However, the period of pentobarbital exposure was short and the extensive subsequent period of heart perfusion prior to cell isolation, cell storage in drug-free KB solution and continual superfusion with barbiturate-free solution in the experimental chamber makes it highly unlikely that murine AVN myocytes were exposed to significant pentobarbital concentration once hearts had been removed and cells isolated. We investigated selected ionic currents in this study and the holding potential of −40 mV used would be anticipated to largely inactivate channels for *I*_Ca,T_, *I*_Na_ and for transient outward K^+^ current, *I*_TO_. This potential limitation is offset, however, by the ability to compare the data from the present study with those from prior rabbit AVN studies that utilized a similar holding potential (Nakayama et* *al. 1984; Choisy et* *al. 2012; Hancox et* *al. 1993; Hancox and Levi 1994b; Convery and Hancox 2000; Cheng et* *al. 2009) and, additionally, with studies of guinea-pig and rat AVN cells that also utilized this approach (Hancox and Levi 1994b; Yuill et* *al. 2010). The principal limitation of the present study is one common to all studies to-date of isolated AVN cells: an inability to attribute with certainty origin from within the AVN of individual isolated cells. Attempts to minimise this limitation have been made for the rabbit by grouping cells by morphology, passive and active electrophysiological properties (Yuill and Hancox 2002; Munk et* *al. 1996; Ren et* *al. 2006, 2008). The present study provides evidence of heterogeneity between murine AVN cells in terms of presence or absence of *I*_f_. Correlating this with prior immunohistochemical data on HCN4 expression (Nikmaram et* *al. 2008; Marger et* *al. 2011b), it is tempting to speculate that Type 1 cells in this study (lacking *I*_f_) may originate from transitional regions whilst Type 2 cells (with *I*_f_) are likely to originate either from the compact AVN or posterior nodal extension. The comparatively small size of the mouse heart presents particular challenges for AVN cell isolation: the landmarks used to identify the region encompassing the AVN lie within an area of approximately 1 mm × 0.6 mm (Zhang et* *al. 2008; Marger et* *al. 2011b), with an estimate from immunohistochemistry data of an area of 0.8 mm × 0.4 mm. It is therefore unlikely that, with current isolation approaches used to isolate AVN cells, it will be possible selectively to isolate cells from different sub-regions of the mouse. Indeed, this would be likely to present challenges for all current small animal models and may be best attempted using hearts from large animal models. Another consequence of the small size of the murine AVN is that cell yields per isolation are small and, at least with our cell isolation technique in its existing form, cells were relatively fragile for electrophysiological recording – particularly when attempting spontaneous AP recording. The difficulty in AP recording precluded both correlation between AP parameters and presence/absence of *I*_f_ and membrane resistance, and an extensive study of ion channel inhibitors on AP parameters. Thus, we focused on basic AP parameter characterization and on the effect of SR inhibition by ryanodine, as this has not previously been reported for murine AVN cells. It is notable that some studies have combined single murine AVN cell voltage clamp with AP recordings using microelectrode measurements from intact tissue (Zhang et* *al. 2008, 2011) a preparation that is likely to be more robust and easier to record from. Although Marger and colleagues have previously recorded murine AVN cell APs (Marger et* *al. 2011a,b), the closer similarity of our data to murine SAN cell APs in terms of AP upstroke velocity and spontaneous AP rates (Lei et* *al. 2004, 2005) suggests to us that future efforts to increase cell viability for AP recording through improvements to our AVN cell isolation technique may be valuable for further AP studies. An alternative or additional approach to electrophysiological AP measurement from mouse single AVN cells that may be of value for future studies is optical measurement of APs using voltage-sensitive dyes; this approach has been of considerable value in studying intact AVN preparations (e.g. [Dobrzynski et* *al. 2003; Efimov et* *al. 2004; Kim et* *al. 2010]).

In conclusion, the present study provides information on murine AVN *I*_f_ that complements existing information on mouse AVN cellular electrophysiology. It provides new information on characteristics of murine AVN *I*_Ca,L_ under conditions comparable to those used previously to study rabbit AVN *I*_Ca,L_, as well as quantitative information on the voltage-dependence of murine AVN *I*_Kr_ that was hitherto lacking. To our knowledge, it also provides the first direct information on *I*_NCX_ and effects of inhibition of SR Ca^2+^ release on murine AVN cell activity. To date, mouse AVN cell computer models that have been made are partially based on extant mouse AVN cell data, but are otherwise based on SAN cell models (Marger et* *al. 2011a; Zhang et* *al. 2011). Data from the present study should both help further development of such models and/or help the future adaptation of rabbit cell models (Inada et* *al. 2009) for the mouse. Importantly, our data implicating a role for Ca^2+^ cycling in murine AVN cell excitability, together with the genetic tractability of the mouse, are suggestive that future work using genetically modified mice will be useful in further elucidating mechanisms of AVN pacemaking.

## Disclosures

None declared.
